# Consensus‐Recommended Follow‐Up Mediates Cardiovascular Outcomes Disparities Between Implantable Cardioverter Defibrillator and Pacemaker Recipients

**DOI:** 10.1111/jce.70521

**Published:** 2026-10-06

**Authors:** Jingwen Huang, Rahul Patel, Chang Liu, Michael S. Lloyd

**Affiliations:** ^1^ Section of Cardiac Electrophysiology, Department of Medicine Emory University School of Medicine Atlanta Georgia USA; ^2^ Department of Epidemiology Emory University Rollins School of Public Health Atlanta Georgia USA

**Keywords:** cardiovascular implantable electronic devices, consensus‐recommended follow‐up, mediation, remote monitoring

## Abstract

**Background:**

Consensus‐recommended follow‐up after cardiac implantable electronic device (CIED) implantation improves outcomes, yet whether it mediates cardiovascular disparities between device types remains unknown.

**Methods:**

We prospectively studied 6806 patients undergoing CIED implantation across a multi‐hospital system (2019–2023; median follow‐up 3.5 years), categorized as implantable cardioverter defibrillator (ICD) (*n* = 2757) or permanent pacemaker (PPM) (*n* = 4049). Causal mediation analysis decomposed the effect of device type on major adverse cardiovascular events (MACE) and device extraction into direct and follow‐up mediated components, adjusting for demographics and comorbidities.

**Results:**

ICD recipients had significantly lower consensus‐recommended follow‐up rates than PPM recipients (65.3% vs. 79.2%; adjusted OR 0.46, 95% CI 0.40–0.52, *p* < 0.001) and substantially higher MACE risk (HR 1.89, 95% CI 1.66–2.25, *p* < 0.001), of which 53.5% was mediated by follow‐up adherence. Device extraction rates were lower in ICD recipients (HR 0.30, 95% CI 0.24–0.38), with only 7.9% mediated by follow‐up adherence.

**Conclusions:**

More than half of the excess cardiovascular risk in ICD recipients was statistically mediated by suboptimal follow‐up adherence—a potentially modifiable pathway. These findings identify follow‐up adherence as a potentially important pathway and support further prospective investigation of strategies to improve monitoring and clinical outcomes in high‐risk CIED populations.

## 1 Introduction

Consensus‐recommended follow‐up after cardiac implantable electronic device (CIED) implantation represents a potentially modifiable factor influencing long‐term outcomes. Prior studies have demonstrated that adherence to recommended CIED follow‐up within 2–12 weeks post‐implantation is associated with improved survival [1], and adherence with remote monitoring has been linked to lower rates of all‐cause mortality and heart failure hospitalizations [2]. However, while the association between consensus‐recommended follow‐up and clinical outcomes has been established, details that may shed light on reasons for this association remain unclear. Specifically, whether consensus‐recommended follow‐up mediates outcome disparities between device types—implantable cardioverter defibrillator (ICD) versus permanent pacemaker (PPM) recipients—has not been investigated. Understanding this mediation relationship has important implications for developing targeted interventions to improve outcomes in high‐risk device populations.

## 2 Methods

We prospectively studied 6806 patients undergoing CIED implantation at a multi‐hospital tertiary care system comprised of four device clinics (2019–2023) with median follow‐up of 3.5 years (IQR 2.2–5.0). Patients were categorized by device type: PPM group (PPM/cardiac resynchronization therapy‐pacemaker [CRT‐P], *n* = 4049) versus ICD group (ICD/CRT‐defibrillator [CRT‐D], *n* = 2757). Consensus‐recommended follow‐up was defined per HRS/EHRA Expert Consensus Class Ia recommendations: for pacemakers, ≥ 1 in‐person visit plus ≥ 1 remote monitoring session; for defibrillators, ≥ 1 in‐person visit plus ≥ 2 remote monitoring sessions within 1‐year post‐implantation [3]. Primary outcomes included major adverse cardiovascular events (MACE: composite of acute myocardial infarction, heart failure hospitalization, stroke, major bleeding, or cardiac arrest) and device extraction (generator removal/exchange or lead removal). MACE was selected to capture clinically meaningful cardiovascular outcomes, while device extraction was included as a CIED‐specific outcome. Logistic and Cox models, adjusted for demographics and comorbidities (hypertension, type 2 diabetes, atrial fibrillation, heart failure, chronic kidney disease, prior myocardial infarction, obesity, and valvular heart disease), assessed consensus‐recommended follow‐up disparities and outcomes. Causal mediation analysis is increasingly used to move beyond simple association and quantify how much of an exposure's effect on an outcome is explained by a specific, measurable pathway versus other unmeasured mechanisms [4, 5]. Mediation analysis was performed using the R package Regression‐Based Causal Mediation Analysis with Interaction and Effect Modification Terms (regmedint) [4], decomposing the total effect of device type on outcomes into indirect effects (mediated through consensus‐recommended follow‐up) and direct effects (from device type to clinical outcomes, independent of follow‐up). The total effect quantifies the overall association between device type and outcome. The proportion mediated was calculated as the ratio of indirect to total effect. The study protocol was approved by the institutional review board, and informed consent was waived. All analyses were performed using R version 4.5.1, with two‐sided *p* < 0.05 considered statistically significant.

## 3 Results

Baseline characteristics differed between groups. ICD recipients were substantially younger (median age 67 vs. 79 years, *p* < 0.001), more males (63.5% vs. 47.6%, *p* < 0.001), and had higher prevalence of heart failure (54.7% vs. 16.5%, *p* < 0.001), prior myocardial infarction (19.9% vs. 8.6%, *p* < 0.001). PPM recipients demonstrated higher prevalence of atrial fibrillation (52.4% vs. 36.5%, *p* < 0.001) and peripheral vascular disease (22.2% vs. 16.0%, *p* < 0.001). ICD recipients had significantly lower odds of meeting consensus‐recommended follow‐up compared to PPM recipients (adjusted OR 0.46, 95% CI 0.40–0.52, *p* < 0.001) after controlling for demographics and comorbidities, with absolute fulfillment rates of 65.3% versus 79.2% (*p* < 0.001). ICD recipients experienced substantially higher MACE risk (hazard ratio [HR] 1.89, 95% CI 1.66–2.25, *p* < 0.001) compared to PPM recipients. Causal mediation analysis revealed that consensus‐recommended follow‐up mediated 53.5% of the elevated MACE risk in ICD recipients (indirect effect HR 1.41, 95% CI 1.37–1.52), with a remaining direct effect of HR 1.34 (95% CI 1.20–1.50) independent of follow‐up adherence (Figure 1A,C). Similarly, ICD recipients had lower device extraction rates (HR 0.30, 95% CI 0.24–0.38, *p* < 0.001), with only 7.9% mediated by consensus‐recommended follow‐up (indirect effect HR 0.91, 95% CI 0.88–0.93; direct effect HR 0.33, 95% CI 0.27–0.42) (Figure 1B,D).

**Figure 1:**
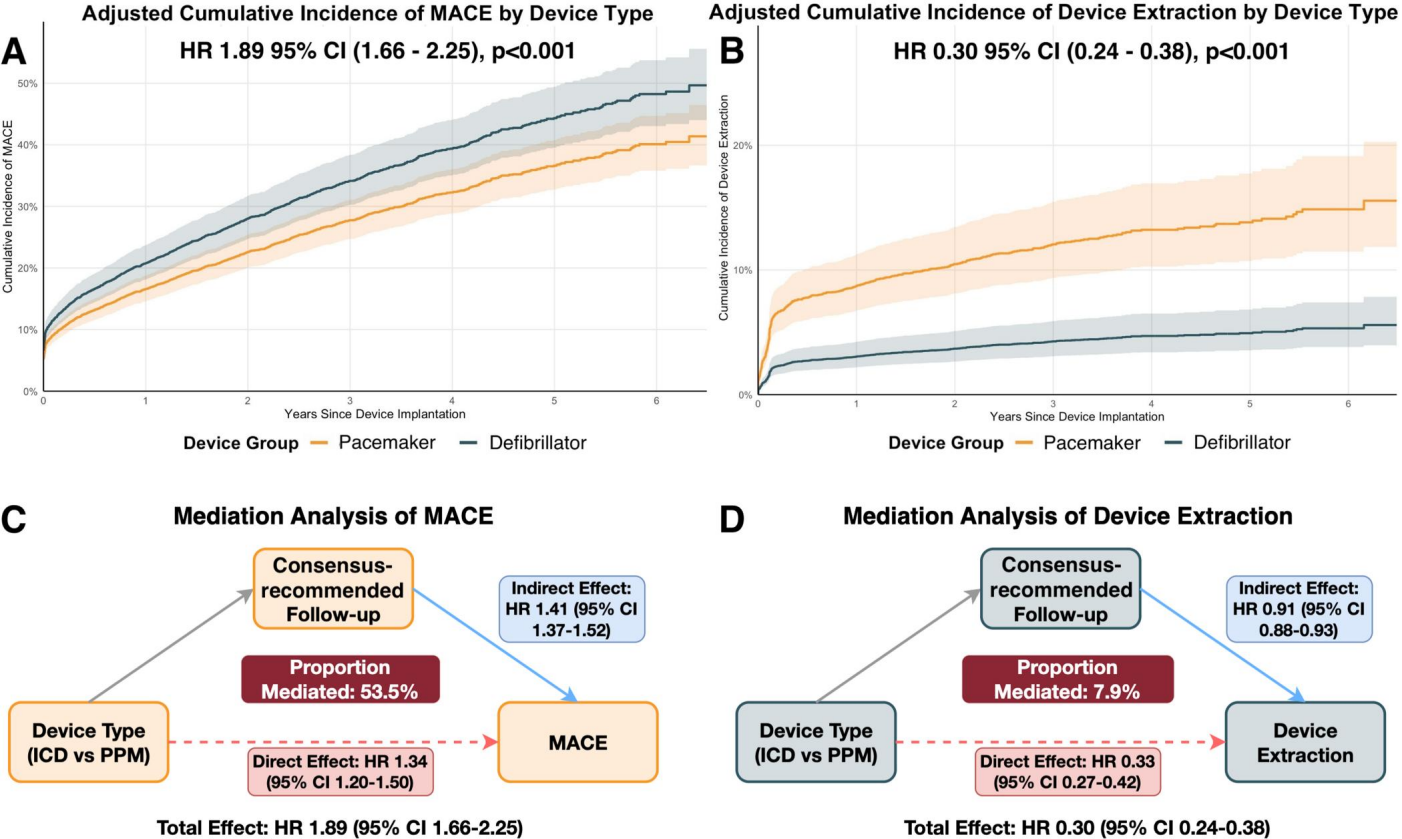
Cumulative incidence and mediation analysis by device type. Panels A and B show adjusted cumulative incidence curves for MACE and device extraction, respectively. Panels C and D present mediation diagrams illustrating how consensus‐recommended follow‐up mediates these device type‐outcome relationships. ICD, implantable cardioverter defibrillator; MACE, major adverse cardiovascular events; PPM, permanent pacemaker.

## 4 Discussion

The findings indicate that more than half of the excess cardiovascular event risk in ICD recipients is associated with suboptimal adherence to consensus‐recommended follow‐up—a potentially modifiable pathway. The indirect effect HR of 1.41 represents the component of excess MACE risk in ICD recipients attributable to their lower consensus‐recommended follow‐up adherence, beyond demographics and comorbidities. Extraction procedures, on the other hand, are driven primarily by device‐specific factors rather than monitoring patterns. The protective effect of ICD type on extraction risk likely reflects differences in lead characteristics and generator longevity, and procedure‐related risks.

These findings have important clinical and health systems implications. The high proportion of mediation for MACE identifies consensus‐recommended follow‐up as a high‐yield intervention target, particularly for ICD populations who face elevated baseline cardiovascular risk and demonstrate lower adherence to recommended monitoring protocols. The lower follow‐up adherence in ICD recipients (65.3% vs. 79.2%) suggests systematic barriers specific to this population. Current remote monitoring technologies and structured follow‐up protocols may help address this gap. These data support the need for device‐specific quality metrics tracking consensus‐recommended follow‐up adherence. Patient navigation programs, technology support initiatives, and systematic identification of barriers to monitoring represent actionable strategies to reduce outcome disparities [6]. Evidence on the benefit of structured device monitoring is mixed [7, 8], with some analyses questioning the economic value of routine follow‐up as currently implemented and a randomized trial of remote monitoring in heart failure failing to show benefit; our findings should therefore be considered hypothesis‐generating.

## 5 Limitations and Conclusion

Study limitations include single‐center design limiting generalizability and observational nature precluding definitive causal inference. This study could not account for potential heterogeneity in programming strategies across participating clinics and providers, which may influence both follow‐up adherence and clinical outcomes. This dataset could not identify the specific clinical actions (e.g., medication or programming changes) occurring at follow‐up encounters responsible for the observed mediation effect. Nonetheless, this mediation analysis identifies consensus‐recommended follow‐up as a potentially important pathway linking device type and cardiovascular outcomes. Prospective studies linking specific follow‐up actions to subsequent outcomes are needed to determine whether improving follow‐up adherence can reduce outcome disparities in high‐risk CIED populations.

## Funding

The authors have nothing to report.

## Conflicts of Interest

The authors declare no conflicts of interest.

## Data Availability

The data that support the findings of this study are available on request from the corresponding author. The data are not publicly available due to privacy or ethical restrictions.
